# Enhanced associations between subjective cognitive concerns and blood-based AD biomarkers using a novel EMA approach

**DOI:** 10.1186/s13195-025-01720-y

**Published:** 2025-04-15

**Authors:** Ángel García de la Garza, Caroline Nester, Cuiling Wang, Jacqueline Mogle, Nelson Roque, Mindy Katz, Carol A. Derby, Richard B. Lipton, Laura Rabin

**Affiliations:** 1 Division of Biostatistics, Department of Epidemiology and Population Health, Albert Einstein College of Medicine, 1300 Morris Park Ave Belfer Bldg 1308B, The Bronx, NY 10461 USA; 2 Saul R. Korey Department of Neurology, Albert Einstein College of Medicine, Bronx, USA; 3 Department of Psychiatry and Human Behavior, Brown University, Providence, USA; 4 Department of Psychology, College of Behavioral, Social and Health Sciences, Clemson University, Clemson, USA; 5 Department of Human Development and Family Studies, Pennsylvania State University, University Park, USA; 6 Department of Psychology, The City University of New York, New York City, USA

**Keywords:** Cognitive concerns, Subjective cognitive decline, Blood-based biomarkers, Alzheimer’s disease, Dementia risk, Ecological momentary assessments

## Abstract

**Background:**

Subjective cognitive concerns (SCC) have emerged as important early indicators of Alzheimer’s disease (AD) risk. Traditional measures of SCC rely on recall-based assessments, which may be limited in capturing real-time fluctuations in cognitive concerns. Ecological Momentary Assessment (EMA) offers a promising alternative by providing real-time data. This study aimed to link SCC assessed via EMA and traditional measures with blood-based AD biomarkers in a diverse, dementia-free, community-based sample based in the Bronx, NY.

**Methods:**

Einstein Aging Study (EAS) participants underwent in-person, recall-based assessments of SCC during an in-clinic visit. Additionally, EMA SCC assessments were collected once per day over two weeks. Linear regressions were conducted to examine the relationships between SCC variables and plasma biomarkers adjusted for demographics and mild cognitive impairment (MCI) status.

**Results:**

In *N* = 254 participants, EMA-reported SCCs demonstrated significant associations with AD biomarkers, particularly p-tau181 (β = 0.21, *p* = 0.001). Further, significant associations remain across both cognitive (cognitively unimpaired vs. MCI) and racial groups. In contrast, traditional SCC measures exhibited limited associations with these biomarkers. The findings highlight the added value of EMA in capturing SCCs that could indicate early ADRD risk.

**Conclusions:**

EMA provides a more dynamic and potentially sensitive method for detecting early AD risk compared to traditional SCC assessments. These real-time measures could enhance early detection and clinical intervention, particularly in diverse and under-resourced populations. This study underscores the potential of EMA for broad applicability and inclusivity in monitoring AD progression and facilitating early therapeutic interventions.

**Supplementary Information:**

The online version contains supplementary material available at 10.1186/s13195-025-01720-y.

## Background

Subjective Cognitive Decline (SCD) represents a pivotal phase within the Alzheimer’s disease (AD) spectrum marked by reporting declines in memory and other aspects of cognition that are not associated with concurrent cognitive deficits on objective neuropsychological tests [1, 2]. SCD often serves as a precursor to more overt deficits, delineating a critical juncture in the progression towards mild cognitive impairment (MCI) and ultimately AD for some older adults [2–4]. Subjective cognitive concerns (SCC) represent the core feature of SCD (e.g., self-perceived cognitive changes, without regard to objective cognitive status of the individual), but they are a non-specific symptom, and may represent subtle objective cognitive difficulties (e.g., due to early Alzheimer’s disease (AD), nutritional deficiencies, sleep disorders, and/or other medical comorbidities) or they be a manifestation of personality traits or depression [5, 6]. SCCs may serve as an accessible and economical screening tool, facilitating the early identification of individuals at heightened risk of neurodegenerative disorders [7, 8]. By leveraging SCC as a potential early indicator, healthcare professionals can work-up, treat, and possibly reverse the causes of self-perceived cognitive change, while implementing timely interventions for ADRD as appropriate [5]. Thus, recognizing SCC not only underscores its importance in the diagnostic framework but also highlights its instrumental value in advancing early intervention strategies for delaying the progression of neurodegenerative diseases.

The best method for assessing SCC is a topic of ongoing debate. Options include single or multiple items capturing one or several domains over various recall intervals [9–11]. Previous studies have shown that the predictive validity of SCC for AD pathology or future progression depends upon how SCC is measured [12–14]. Moreover, the assessment of SCC and its correlation with future dementia risk is confounded by variations in demographic factors such as race, gender, age, and ethnoracial disparities [15–17], thereby complicating efforts to optimally quantify SCCs.

While traditional assessments, typically administered in clinical settings using pen and paper, are valuable for measuring cognitive concerns, they may not fully capture the natural variability in daily cognition and are prone to recall bias [18–20] underscoring the need for additional assessment methods that capture more ecologically relevant aspects of daily cognition. Asking someone with possible memory impairment to characterize their memory at present and compare it with their performance months or years earlier is a challenging task. Smartphone-based Ecological Momentary Assessment (EMA) emerges as a promising solution by capturing real-time perception of cognitive function over a brief interval as individuals engage in their daily activities [21, 22]. This approach offers dynamic, contextually relevant data to complement information gathered from traditional assessment methods, helping to bridge the gap between subjective cognitive lapses and objective clinical evaluations [19, 20, 23]. EMA also greatly reduces the recall period for cognitive lapses reducing recall bias.

Blood-Based Biomarkers of AD offer a non-invasive, cost-effective solution for early detection and disease monitoring [24, 25]. Blood-Based biomarkers, such as p-tau181, Aβ40, Aβ42 as well as their ratio (Aβ42/ Aβ40) are associated with prospective cognitive decline [26, 27] future conversion to MCI [28–31] and further conversion to AD [32–36]. Beyond diagnosis, they enhance accessibility as an alternative to PET imaging in aging research [33, 37]. Previous research in SCC and blood-based AD biomarkers has primarly focused on blood biomarkers as indicators of increased AD risk or underlying AD pathology in individuals with SCD [38, 39]. Emerging evidence suggests that both p-tau181 and Aβ42/ Aβ40 in individuals with SCD help predict clinical progression progression to MCI or AD [6, 40, 41]. Studies have shown that p-tau181 can discriminate AD pathology identified through PET or CSF in individuals with SCD [42–45]. Further, increased levels of plasma NfL have also been reported in SCD and CU individuals [46], there is limited research directly examining the associations between SCCs and blood-based AD biomarkers. We identified one study that investigated the association between SCC and blood-based biomarkers that showed that greater informant reported cognitive change, but not self-reported cognitive change, was associated with higher levels of plasma p-tau181 [47]. 

Findings from this emerging literature linking SCC and blood-based AD biomarkers suggest potential avenues for new research, including the development of better SCC measures to serve as scalable indicators of AD pathology, progression, and disease monitoring. One of the benefits of SCC assessment, particularly via smartphone-based EMA, is that it can be implemented remotely in rural or under-resourced settings, thus expanding access to AD risk screening. Unfortunately, studies on SCC and AD biomarkers that include ethnic/racially diverse participants are presently lacking [38, 48]. This is of urgent concern, as minoritized groups are at increased risk for AD, face increased burden of SCC [49], may be less likely to report these concerns to health care providers if unprompted [38], and face double the risk of dementia misdiagnosis [15–17]. AD blood-based biomarkers alone may lack sufficient specificity for definitive AD diagnosis or progression risk stratification, necessitating complementary symptom-based assessments such as SCCs. It is therefore possible that early and accurate diagnosis, treatment, and clinical care could be enhanced for minoritized and underserved groups through careful assessment of SCC in tandem with blood-based AD biomarkers, which are relatively low cost, low effort, and highly scalable techniques.

Our aim is to evaluate the potential use of SCC to serve as an indicator of early AD pathology by examining the link between SCC, ascertained via both EMA and traditional, in-person paper-and-pencil measures, and blood-based AD biomarkers in a demographically diverse community sample without dementia. The Einstein Aging Study provides an ideal cohort for this work because it includes a diverse sample of community-dwelling adults. This diversity allows us to determine whether associations between SCC and biomarkers hold in the general community, not just in predominantly non-Hispanic White samples recruited from memory clinics. Additionally, the study measures SCC using both EMA and paper-and-pencil instruments. This dual approach enables direct comparisons of different SCC measurements and how these measurement methods might impact the association with AD biomarkers.

Herein, we test (1) whether self-reported SCCs, measured by EMA and traditional SCC measures, are associated with increased levels of plasma AD biomarkers; (2) whether these associations hold when stratifying by objective neuropsychological status (e.g., neuropsychologically unimpaired versus mild cognitive impairment); and (3) whether there are any differences when stratifying by self-identified racial background.

## Methods

### Participants

Participants were recruited from the Einstein Aging Study (EAS), a longitudinal cohort study of older adults in Bronx County, NY. Participants are identified through systematic sampling from the registered voter lists of the Bronx, followed by preliminary screening via phone to determine eligibility. Eligibility criteria include being at least 70 years old, ambulatory, a Bronx resident, non-institutionalized, English-speaking, and without visual or auditory impairments that would hinder neuropsychological testing. Additionally, participants must not have active psychiatric symptoms that interfere with assessment completion [50] or prevalent dementia, as determined by the telephone version of the Memory Impairment Screen (MIS) [51]. Participants engage in annual in-person study visits, which encompass assessments of comorbidities, medication use, clinical neurological exams, neuropsychological assessment, anthropometric measures, visual inspection of medications and fasting blood draws. Informed consent is obtained in accordance with protocols approved by the local institutional review board. At enrollment and at annual follow-up visits participants complete an in-person clinic assessment and a 14-day smartphone-based EMA protocol. The Cognitive Change Index (CCI), which is a widely used paper-and pencil SCC questionnaire [52, 53], was given on day one of the in-person visit. Participants returned a week later for a second visit to start the EMA protocol described below. Our cross-sectional analyses include baseline data collected between May 2017 and January 2020.

### Alzheimer’s disease blood biomarkers

Fasting blood samples were collected in EDTA-coated collection tubes. Following centrifugation, plasma was separated from the cell pellet, aliquoted into 0.5 mL cryo tubes and stored at −80C pending biomarker measurement. Plasma Aβ40, Aβ42, NfL and GFAP concentrations were measured by Single molecule array (Simoa) technology using the NEUROLOGY 4-PLEX E assay on an HD-X instrument as described by the manufacturer (Quanterix, Billerica, MA). Plasma p-tau181 concentration was measured using an in-house Simoa assay as previously described in detail [27]. All measurements were performed in singlicates in one round of experiments using one batch of reagents. Intra-assay coefficients of variation, monitored using duplicate quality control samples in the beginning and end of each plate, were < 10%.

### In-clinic assessments of SCC

As noted, participants completed a widely used paper-and pencil SCC measure (i.e., 40-item version of the CCI) at the in-person study visit. The original CCI includes 20 items that assess self-perceived ability in memory and other cognitive domains compared to 5 years ago. The 40-item version (i.e., CCI-40) includes an additional 20 items and the overall measures probes concerns across various domains, including memory, executive functioning, language, attention/concentration, visuospatial, mental clarity/efficiency, orientation, and calculation [52]. Sample CCI rubric and items are presented in Supplement Table 1.

**Table 1:** Demographics

Characteristic	*N* = 254^1^
Gender	
Female	170 (67%)
Male	84 (33%)
Race / Ethnicity	
Non-Hispanic White	124 (49%)
Non-Hispanic Black	103 (41%)
Hispanic	27 (11%)
Years of Education	15.19 (3.65)
Age	77.40 (4.85)
Cognitive Status	
Cognitively Unimpaired	179 (70%)
Mild Cognitive Impairment	75 (30%)
DMLC Total Score	1.46 (1.62)
DMLC Memory Score	0.97 (1.02)
DMLC Non-Memory Score	0.49 (0.73)
Number of DMLC Days Completed	11.03 (2.81)
CCI-20 Total Score	34.34 (11.38)
CCI-20 Memory Score	22.51 (8.00)
CCI-20 Not-Memory Score	11.83 (4.29)

^*1*^ n / N (%); Mean (SD)

**Table 2:** Associations between each SCC metric and biomarker on the entire sample (*N* = 254), adjusted for the following covariates: age, gender, race/ethnicity, cognitive status (MCI/CU), years of education, and depression (as measured by the geriatric depression Scale). Bold indicates significant association at $\:\alpha\:=0.05$

SCC Metric	Aβ40		Aβ42		GFAP		NfL		*p*-tau181		Aβ42 / Aβ40	
	Beta (95% CI)^1^	*p*-value	Beta (95% CI)^1^	*p*-value	Beta (95% CI)^1^	*p*-value	Beta (95% CI)^1^	*p*-value	Beta (95% CI)^1^	*p*-value	Beta (95% CI)^1^	*p*-value
DMLC Total	0.07 (-0.04 to 0.17)	0.226	0.00 (-0.12 to 0.11)	0.946	-0.01 (-0.14 to 0.11)	0.823	0.03 (-0.07 to 0.12)	0.569	0.21 (0.08 to 0.33)	**0.001**	-0.11 (-0.23 to 0.00)	0.056
DMLC Memory	0.08 (-0.02 to 0.19)	0.125	0.02 (-0.10 to 0.14)	0.738	0.00 (-0.13 to 0.12)	0.939	0.06 (-0.04 to 0.16)	0.216	0.23 (0.10 to 0.35)	**< 0.001**	-0.10 (-0.22 to 0.02)	0.092
DMLC Not-Memory	0.03 (-0.08 to 0.14)	0.58	-0.04 (-0.15 to 0.08)	0.544	-0.03 (-0.15 to 0.10)	0.695	-0.02 (-0.12 to 0.07)	0.656	0.14 (0.02 to 0.27)	**0.026**	-0.11 (-0.23 to 0.00)	0.059
CCI-20 Total	0.00 (-0.11 to 0.12)	0.964	-0.08 (-0.20 to 0.04)	0.186	-0.02 (-0.16 to 0.12)	0.764	0.03 (-0.07 to 0.13)	0.556	0.13 (-0.01 to 0.26)	0.061	-0.12 (-0.25 to 0.00)	0.052
CCI-20 Memory	-0.02 (-0.13 to 0.09)	0.73	-0.10 (-0.22 to 0.02)	0.119	-0.03 (-0.17 to 0.10)	0.629	0.02 (-0.08 to 0.12)	0.706	0.12 (-0.01 to 0.26)	0.07	-0.09 (-0.22 to 0.03)	0.127
CCI-20 Not Memory	0.04 (-0.07 to 0.16)	0.438	-0.03 (-0.16 to 0.09)	0.585	0.01 (-0.13 to 0.14)	0.903	0.04 (-0.06 to 0.15)	0.397	0.11 (-0.03 to 0.24)	0.128	-0.14 (-0.27 to -0.02)	**0.024**

^*1*^ CI = Confidence Interval

Our main analysis focuses on the total score of the CCI-20 item version [53] as it is the most widely cited in the literature [10, 11]. Total scores on the CCI range from 20 to 100, with higher scores indicating worse cognitive difficulties or change. Participants (*N* = 4) who did not answer all CCI items were excluded from the analyses. Similar to our approach with the EMA items, we also report associations with all memory items in the CCI-20 (referred to as ‘CCI-20 Memory,’ equivalent to the CCI-12 used in the Alzheimer’s Disease Neuroimaging Initiative, ADNI), as well as the non-memory items in the CCI-20 (referred to as ‘CCI-20 Non-Memory’), including executive functioning and language items. Scores on the CCI-20 Memory range from 12 to 60, and scores in the CCI-20 non-Memory range from 8 to 40. Thus, in supplemental analyses, we were able to capture all extant versions of the CCI (i.e., CCI-20, CCI-40, and CCI-12)—as there is currently no consensus as to which version is optimal.

### Smartphone assessment of subjective cognitive concerns

At the second study visit, participants received a training session on using the smartphone to answer the EMA-based surveys. Participants were also given two additional days to practice using the smartphone before the start of the EMA protocol. During the 14-day daily diary assessment protocol [54], participants completed the Daily Memory Lapses Check (DMLC) [23, 54] to document real-time occurrences of cognitive lapses in memory, executive functioning, and other domains. Each evening, participants self-initiated a smartphone survey to report their SCC lapses. The novel DMLC questionnaire is presented in the supplement. We summarize responses by computing the average number of SCC lapses per day during those days during the sampling period in which the DMLC was completed. These calculations are applied to the overall DMLC (comprising 17 items, variables: DMLC total), as well as within specific cognitive domains such as memory (retrospective and prospective; comprising 10 items, variables: DMLC memory total) and other cognitive functions (comprising 7 items, variables: DMLC non-memory total), including executive functioning, processing speed, attention, and visuospatial abilities. Higher averages indicate more SCCs lapses in daily life. The scores for the DMLC Total range between 0 and 21. The scores for the DMLC Memory range between 0 and 14. The scores for the DMLC Non-Memory range between 0 and 7.

Participants needed to have completed at least three assessments during the 14-day sampling period to be included in the present analysis. The DMLC Smartphone App allowed participants to complete the evening survey more than once. In cases where participants had completed more than one evening survey, we opted to include data only from the first completion (i.e., the earliest one after the prompt), as we believe this is the most similar observation to the remainder of the 14-day protocol. Notably, we reran all the analyses presented in this manuscript excluding data from days in which participants completed the evening survey more than once (see Sensitivity Analysis 2 in Supplement). This excludes one further participants from the study, who no longer had 3 or more days of data, and the results remain unchanged.

### Mild cognitive impairment and cognitively unimpaired classifications

Participants were classified as either having MCI or being cognitively/ neuropsychologically unimpaired (CU) based on the Jak/Bondi criteria [55, 56], utilizing data from in person neuropsychological test performance at the in-person EAS clinic visit [57]. We used Jak/Bondi criteria for MCI, which did not require SCC and reducing the potential for circularity which might arise using other definitions of MCI. The classification utilized 10 neuropsychological tools spanning five cognitive domains: (1) Memory: Free and Cued Selective Reminding Test [Free recall] [58], Benson Complex Figure [Delayed recall]; [59] (2) Executive Function: Trail Making Test Part B [time limit 300 s] [60], Phonemic Verbal Fluency [Letters F, L for 1 min each]; [61] (3) Attention: Trail Making Test Part A [time limit 150 s] [60], Number Span [forward and backward sequences] [62], (4) Language: Multilingual Naming Test [MINT, total score] [63], Category Fluency [Animals, Vegetables: 1 min each]; [64] (5) Visual-spatial: Benson Complex Fig [59]., WAIS III Block Design [62]. Classification was based on the following actuarial criteria: (1) impaired scores, defined as > 1 SD below the normative means adjusted for age, gender, and education, in two tests within at least one cognitive domain (e.g., memory, language, or speed/executive function); or (2) one impaired score, defined as > 1 SD below the normative means, in each of three of the five domains. If neither of these criteria were met, the individual was classified as CU. Given that SCC was the primary variable/predictor of interest in this study, both MCI and CU were diagnosed independent of SCC reporting. As such, we treat level of SCC endorsement as a continuous predictor, regardless of objective neuropsychological status, rather than utilizing SCC to classify a diagnostic category (i.e., as in the condition of SCD).

### Statistical analysis

We summarized baseline demographic and health-related information for the entire sample, which included data on age, sex, race/ethnicity, years of education, SCC smartphone metrics (DMLC Total, DMLC Memory Total, DMLC Non-Memory Total), SCC pen-and-paper metrics (CCI-20, CCI-20 Memory, CCI-20 Non-Memory), and the presence of MCI. We further summarized the number of completed DMLC assessments. Next, we conducted separate linear regressions for each of the outcome plasma biomarkers as the outcome: Aβ40, Aβ42, the Aβ40/Aβ42 ratio, p-tau181, Neurofilament Light Chain (NfL), and Glial Fibrillary Acidic Protein (GFAP). The goal of these regression analyses was to explore the cross-sectional associations between AD plasma biomarkers and SCC variables while adjusting for age, sex, years of education, Depression (assessed through the Geriatric Depression Scale excluding the single memory item), and MCI status. We analyzed both the DMLC SCCs (DMLC Total, DMLC Memory Total, DMLC Non-Memory Total) and SCC pen-and-paper metrics (CCI-20, CCI-20 Memory, CCI-20 Non-Memory). DMLC and CCI-20 scores were standardized for consistency and easier interpretation across variables. Biomarkers were also standardized, with two extreme outliers (10 SD above the mean) removed.

As we are interested in the association of AD biomarkers and SCCs within each cognitive status group and race/ethnicity, we also conducted another set of linear regressions where we stratified by MCI or CU status instead of adjusting for MCI status, and a third set of regressions stratified by race/ethnicity (non-Hispanic White, non-Hispanic Black) rather than adjusting for race. As the number of Hispanic participants is small, we focused exclusively on NH-W and NH-B participants when stratifying by race. The aim was to identify whether the cross-sectional associations between AD plasma biomarkers and SCC hold within each of these subgroups.

## Results

### Overview of the sample

**Fig. 1:**
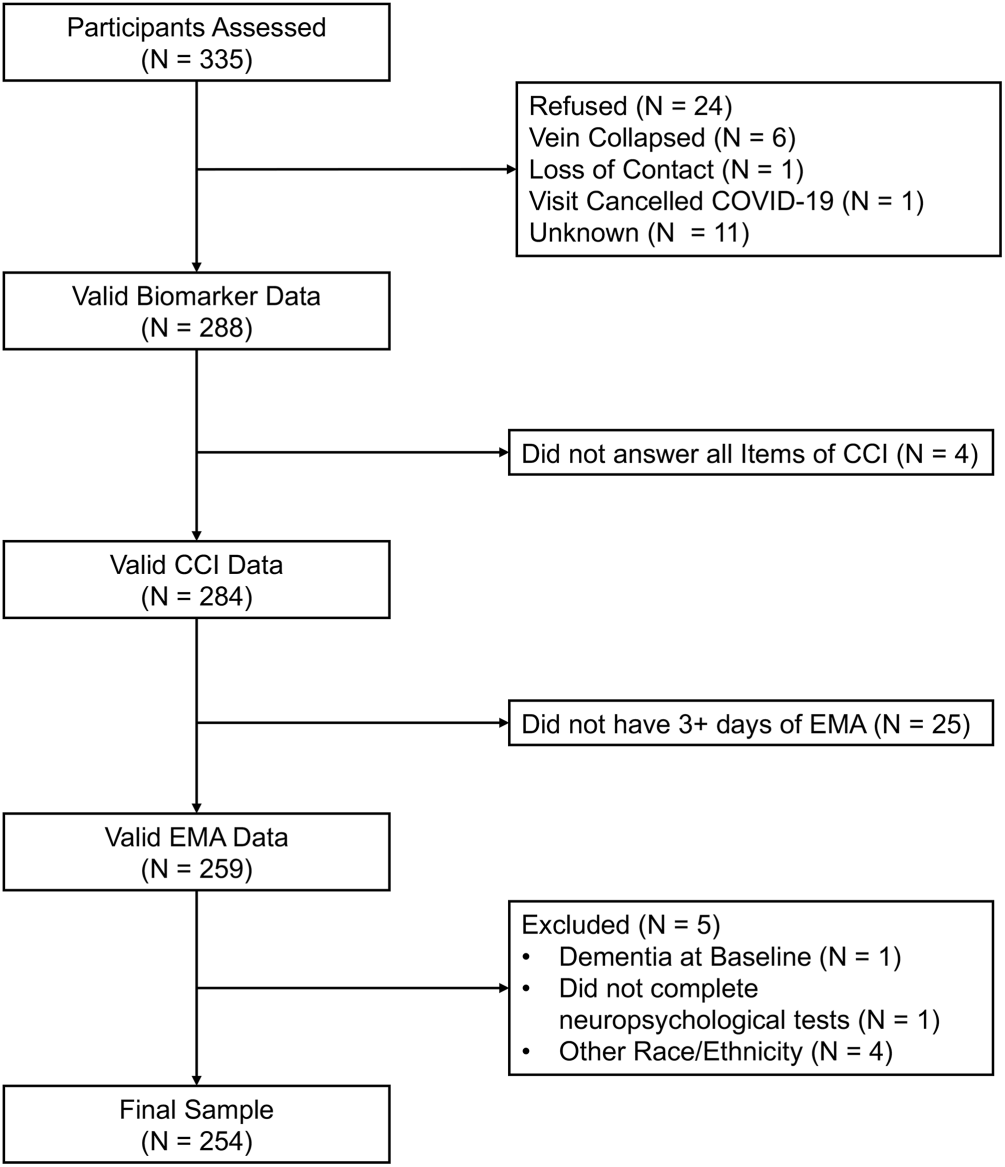
Flow Diagram of the Einstein Aging Study Sample

Among EAS participants assessed between May 2017 and January 2020, this analysis is based on *n* = 254 who provided a fasting blood sample, completed at least 3 days of EMA, completed the CCI questionnaire and the neuropsychological assessments, were free of dementia, and indicated that their race/ethnicity was White or Black (Fig. 1). We present a summary of participant’s demographics in Table 1. Participants’ ages ranged from 70 to 91 years, with a mean age of 77.4 years (SD = 4.8). The sample comprised 67% women, with an average educational attainment of 15.2 ± 3.7 years. Among the participants, 48.8% identified as Non-Hispanic White (NH-W), 40.6% as Non-Hispanic Black (NH-B), and 10.6% as Hispanic. Out of the entire sample, 75 (29.5%) participants met the Jak/Bondi criteria for MCI. Across the entire sample, the DMLC Total Score correlated with the Total CCI-20 (ρ = 0.52, *p* < 0.001). Similarly, DMLC Memory scores correlated with the CCI-20 Memory Total (*r* = 0.47, *p* < 0.001), and DMLC Non-Memory scores correlated with the CCI-20 Non-Memory Total (*r* = 0.48, *p* < 0.001). The average number of days participants answered the DMLC was 11.03 ± 2.81 out of 14. Participants reported an average of 1.46 ± 1.62 daily SCCs in the DMLC, with the majority being memory-related (0.97 ± 1.02) and the rest being non-memory (0.49 ± 0.73) SCCs. The average score on the CCI-20 was 34.34 ± 11.38. The average score on the memory items of the CCI-20 was 22.51 ± 8.00, and the average score on the non-memory items of the CCI-20 was 11.83 ± 4.29.

### Associations of SCC and biomarkers in entire sample

Results from separate linear regressions testing the association of each SCC variable with each biomarker, adjusted for age, sex, MCI status, years of education, and race/ethnicity are presented in Table 2. There was a significant association between p-tau181 and DMLC Average scores (β = 0.21, *p* = 0.001), DMLC Memory concerns (β = 0.23, increase *p* < 0.001), and a DMLC Non-Memory Concerns (β = 0.14, *p* = 0.026). However, no significant associations were found between the CCI-20 Total, CCI-20 Memory, and CCI-20 Non-Memory Scores and p-tau181. We found a negative association between the CCI-20 Non-Memory Score and Aβ42/Aβ40 ratio (β = -0.14, *p* = 0.024). No other associations between SCCs were detected with any of Aβ40, Aβ42, NfL, or GFAP.

### Associations of SCC and biomarkers stratified by MCI / CU subgroup

We identified unique relationships between biomarkers and SCC within both the MCI and CU groups (see Table 3). Specifically, the DMLC Total score was associated with p-tau181 in the CU individuals (β = 0.20, *p* = 0.01) but not in the MCI group. Furthermore, in CU individuals, we found an association between the DMLC Memory score and p-tau181 (β = 0.25, *p* < 0.001), which was not found in those with MCI. Conversely, the DMLC Non-Memory score correlated with p-tau181 solely in the MCI group (β = 0.27, *p* = 0.024). Interestingly, no significant association was observed between the CCI-20 Total, CCI-20 Memory, and CCI-20 Not Memory Scores and p-tau181 in either MCIs or CU individuals.

**Table 3:** Associations between each SCC metric and biomarker stratified by cognitive status (MCI / CU), adjusted for the following covariates: age, gender, race/ethnicity, years of education, and depression (as measured by the geriatric depression Scale). Bold indicates significant association at $\:\alpha\:=0.05$

Mild Cognitive Impairment (*N* = 75)												
**SCC Metric**	Aβ40		Aβ42		GFAP		NfL		p-tau181		Aβ42 / Aβ40	
	**Beta** **(95% CI)** ^*1*^	**p-value**	**Beta** **(95% CI)** ^*1*^	**p-value**	**Beta** **(95% CI)** ^*1*^	**p-value**	**Beta** **(95% CI)** ^*1*^	**p-value**	**Beta** **(95% CI)** ^*1*^	**p-value**	**Beta** **(95% CI)** ^*1*^	**p-value**
DMLC Total	0.30 (0.12 to 0.48)	**0.001**	0.09 (-0.13 to 0.31)	0.426	0.13 (-0.11 to 0.37)	0.275	0.10 (-0.03 to 0.22)	0.141	0.22 (-0.01 to 0.46)	0.065	-0.33 (-0.56 to -0.10)	**0.006**
DMLC Memory	0.30 (0.12 to 0.48)	**0.001**	0.11 (-0.11 to 0.33)	0.329	0.07 (-0.17 to 0.31)	0.542	0.08 (-0.04 to 0.21)	0.195	0.18 (-0.06 to 0.42)	0.144	-0.30 (-0.53 to -0.06)	**0.014**
DMLC Not-Memory	0.25 (0.07 to 0.44)	**0.007**	0.05 (-0.17 to 0.26)	0.677	0.21 (-0.02 to 0.44)	0.079	0.10 (-0.02 to 0.23)	0.111	0.27 (0.04 to 0.50)	**0.024**	-0.34 (-0.57 to -0.11)	**0.004**
CCI-20 Total	0.19 (0.00 to 0.39)	0.052	-0.08 (-0.30 to 0.15)	0.511	-0.09 (-0.33 to 0.16)	0.494	0.05 (-0.09 to 0.18)	0.466	0.23 (-0.01 to 0.48)	0.061	-0.40 (-0.64 to -0.17)	**0.001**
CCI-20 Memory	0.16 (-0.03 to 0.36)	0.099	-0.10 (-0.33 to 0.12)	0.362	-0.10 (-0.34 to 0.15)	0.44	0.02 (-0.11 to 0.16)	0.732	0.21 (-0.03 to 0.46)	0.087	-0.38 (-0.62 to -0.14)	**0.002**
CCI-20 Not Memory	0.21 (0.01 to 0.40)	**0.036**	-0.01 (-0.24 to 0.22)	0.945	-0.05 (-0.30 to 0.20)	0.694	0.09 (-0.05 to 0.22)	0.192	0.23 (-0.02 to 0.47)	0.072	-0.37 (-0.61 to -0.13)	**0.003**

**Cognitively Unimpaired (*N*** **= 179)**												
**SCC Metric**	Aβ40		Aβ42		GFAP		NfL		p-tau181		Aβ42 / Aβ40	
	**Beta** **(95% CI)** ^*1*^	**p-value**	**Beta** **(95% CI)** ^*1*^	**p-value**	**Beta** **(95% CI)** ^*1*^	**p-value**	**Beta** **(95% CI)** ^*1*^	**p-value**	**Beta** **(95% CI)** ^*1*^	**p-value**	**Beta** **(95% CI)** ^*1*^	**p-value**
DMLC Total	-0.05 (-0.18 to 0.08)	0.468	-0.04 (-0.18 to 0.09)	0.545	-0.09 (-0.24 to 0.06)	0.234	-0.01 (-0.13 to 0.12)	0.895	0.20 (0.05 to 0.34)	**0.01**	0.01 (-0.12 to 0.14)	0.901
DMLC Memory	-0.03 (-0.16 to 0.10)	0.623	-0.02 (-0.16 to 0.12)	0.773	-0.06 (-0.21 to 0.10)	0.477	0.05 (-0.08 to 0.17)	0.468	0.25 (0.10 to 0.40)	**< 0.001**	0.02 (-0.11 to 0.15)	0.762
DMLC Not-Memory	-0.06 (-0.19 to 0.07)	0.389	-0.06 (-0.20 to 0.08)	0.383	-0.12 (-0.27 to 0.03)	0.126	-0.07 (-0.20 to 0.05)	0.245	0.09 (-0.06 to 0.24)	0.25	-0.01 (-0.14 to 0.12)	0.895
CCI-20 Total	-0.10 (-0.24 to 0.04)	0.145	-0.09 (-0.23 to 0.06)	0.249	0.01 (-0.15 to 0.18)	0.862	0.02 (-0.12 to 0.16)	0.773	0.05 (-0.11 to 0.21)	0.551	0.04 (-0.10 to 0.18)	0.592
CCI-20 Memory	-0.11 (-0.24 to 0.03)	0.132	-0.08 (-0.22 to 0.07)	0.288	0.00 (-0.16 to 0.16)	0.995	0.02 (-0.12 to 0.15)	0.791	0.06 (-0.10 to 0.22)	0.434	0.06 (-0.08 to 0.20)	0.403
CCI-20 Not Memory	-0.07 (-0.21 to 0.07)	0.334	-0.08 (-0.23 to 0.07)	0.31	0.04 (-0.13 to 0.21)	0.647	0.02 (-0.12 to 0.16)	0.8	0.01 (-0.16 to 0.17)	0.944	-0.01 (-0.16 to 0.13)	0.842

^*1*^ CI = Confidence Interval

In terms of plasma Aβ40, we found associations with most SCC variables in the MCI group: DMLC Total score (β = 0.30, *p* = 0.001), DMLC Memory score (β = 0.30, *p* = 0.001), DMLC Non Memory score (β = 0.25, *p* = 0.007), and CCI-20 Non Memory (β = 0.21, *p* = 0.036). However, no such associations were observed in the CU group. Additionally, all SCC variables were negatively associated with the Aβ42/Aβ40 ratio in the MCI group but not in the CU: DMLC Total (β = -0.33, *p* = 0.006), DMLC Memory (β = -0.30, *p* = 0.014), DMLC Non Memory (β = -0.34, *p* = 0.004), CCI-20 Total (β = -0.40, *p* = 0.001), CCI-20 Memory (β = -0.38, *p* = 0.002), and CCI-20 Non-Memory (β = -0.37, *p* = 0.003). No associations between SCCs were detected with Aβ42, NfL, or GFAP.

### Associations of SCC and biomarkers stratified by race

We focus on non-Hispanic Whites and non-Hispanic Blacks only due to the limited number of Hispanics in our study. Our stratified analysis indicated that the method of SCC collection’s sensitivity to biomarkers differed within each race/ethnicity group (Table 4). Specifically, the DMLC Total score was associated with p-tau181 in the NH-W (β = 0.31, *p* = 0.003), and so was the DMLC Memory score (NH-W: β = 0.34, *p* < 0.001), but neither was significant in the NH-B. Conversely, the CCI-20 Total score was associated with p-tau181 in the NH-B (β = 0.19, *p* = 0.04), and so is the CCI-20 Memory score (β = 0.20, *p* = 0.03), but neither was significant in the NH-W. We found that the CCI-20 Not-Memory Score was negatively associated with Aβ42/Aβ40 ratio in the NH-B (β = -0.20, *p* = 0.034) but not in the NH-Ws.

**Table 4:** Associations between each SCC metric and biomarker stratified by Race / Ethnicity (NH-White & NH-Black) adjusted for the following covariates: age, gender, cognitive status (MCI / CU), years of education, and depression (as measured by the geriatric depression Scale). Bold indicates significant association at $\:\alpha\:=0.05$

Non-Hispanic Black (*N* = 103)												
**SCC Metric**	Aβ40		Aβ42		GFAP		NfL		p-tau181		Aβ42 / Aβ40	
	**Beta** **(95% CI)** ^*1*^	**p-value**	**Beta** **(95% CI)** ^*1*^	**p-value**	**Beta** **(95% CI)** ^*1*^	**p-value**	**Beta** **(95% CI)** ^*1*^	**p-value**	**Beta** **(95% CI)** ^*1*^	**p-value**	**Beta** **(95% CI)** ^*1*^	**p-value**
DMLC Total	0.06 (-0.09 to 0.22)	0.414	-0.03 (-0.20 to 0.14)	0.76	0.11 (-0.09 to 0.31)	0.284	0.02 (-0.12 to 0.16)	0.788	0.16 (-0.02 to 0.34)	0.084	-0.16 (-0.34 to 0.02)	0.074
DMLC Memory	0.07 (-0.09 to 0.23)	0.369	-0.01 (-0.18 to 0.17)	0.95	0.11 (-0.09 to 0.30)	0.295	0.00 (-0.14 to 0.14)	0.977	0.18 (-0.01 to 0.36)	0.057	-0.14 (-0.31 to 0.04)	0.131
DMLC Not-Memory	0.05 (-0.11 to 0.20)	0.559	-0.05 (-0.22 to 0.12)	0.544	0.09 (-0.10 to 0.29)	0.349	0.04 (-0.10 to 0.18)	0.558	0.11 (-0.07 to 0.29)	0.232	-0.17 (-0.34 to 0.00)	0.054
CCI-20 Total	0.03 (-0.13 to 0.19)	0.748	-0.11 (-0.28 to 0.07)	0.228	0.03 (-0.17 to 0.24)	0.746	-0.01 (-0.15 to 0.14)	0.938	0.19 (0.01 to 0.38)	**0.04**	-0.18 (-0.36 to 0.00)	0.052
CCI-20 Memory	-0.02 (-0.18 to 0.14)	0.81	-0.14 (-0.31 to 0.03)	0.101	0.02 (-0.18 to 0.23)	0.812	-0.04 (-0.18 to 0.11)	0.605	0.20 (0.02 to 0.38)	**0.032**	-0.15 (-0.33 to 0.03)	0.105
CCI-20 Not Memory	0.11 (-0.05 to 0.27)	0.188	-0.01 (-0.19 to 0.17)	0.9	0.04 (-0.16 to 0.25)	0.678	0.06 (-0.09 to 0.21)	0.442	0.14 (-0.05 to 0.32)	0.159	-0.20 (-0.38 to -0.02)	**0.034**

**Non-Hispanic White (*N*** **= 124)**												
**SCC Metric**	Aβ40		Aβ42		GFAP		NfL		p-tau181		Aβ42 / Aβ40	
	**Beta** **(95% CI)** ^*1*^	**p-value**	**Beta** **(95% CI)** ^*1*^	**p-value**	**Beta** **(95% CI)** ^*1*^	**p-value**	**Beta** **(95% CI)** ^*1*^	**p-value**	**Beta** **(95% CI)** ^*1*^	**p-value**	**Beta** **(95% CI)** ^*1*^	**p-value**
DMLC Total	0.12 (-0.05 to 0.29)	0.166	0.04 (-0.14 to 0.21)	0.668	-0.08 (-0.28 to 0.12)	0.428	0.02 (-0.14 to 0.18)	0.777	0.31 (0.11 to 0.50)	**0.003**	-0.13 (-0.31 to 0.05)	0.168
DMLC Memory	0.15 (-0.02 to 0.32)	0.076	0.07 (-0.10 to 0.25)	0.426	-0.05 (-0.25 to 0.15)	0.617	0.10 (-0.06 to 0.26)	0.207	0.34 (0.14 to 0.54)	**< 0.001**	-0.12 (-0.30 to 0.06)	0.176
DMLC Not-Memory	0.05 (-0.12 to 0.22)	0.536	-0.01 (-0.18 to 0.16)	0.897	-0.10 (-0.30 to 0.10)	0.321	-0.08 (-0.23 to 0.08)	0.327	0.20 (0.00 to 0.40)	0.05	-0.10 (-0.28 to 0.07)	0.253
CCI-20 Total	-0.04 (-0.22 to 0.13)	0.634	-0.05 (-0.23 to 0.13)	0.577	-0.10 (-0.31 to 0.10)	0.328	0.03 (-0.13 to 0.19)	0.71	0.00 (-0.21 to 0.21)	0.986	0.03 (-0.16 to 0.21)	0.755
CCI-20 Memory	-0.04 (-0.21 to 0.13)	0.64	-0.05 (-0.23 to 0.13)	0.576	-0.13 (-0.33 to 0.08)	0.22	0.04 (-0.12 to 0.20)	0.658	-0.02 (-0.23 to 0.19)	0.832	0.03 (-0.16 to 0.21)	0.772
CCI-20 Not Memory	-0.03 (-0.20 to 0.14)	0.735	-0.03 (-0.21 to 0.14)	0.708	-0.02 (-0.22 to 0.19)	0.876	0.01 (-0.15 to 0.17)	0.915	0.05 (-0.16 to 0.26)	0.63	0.02 (-0.16 to 0.21)	0.798

^*1*^ CI = Confidence Interval

### Supplementary analyses

As a supplementary sensitivity analysis, we estimated the associations of CCI-40 to AD biomarkers and compared results to those found using the CCI-20. Results using the CCI-20 and CCI-40 were similar. Although the CCI-40 appeared to be more sensitive to biomarkers. The CCI-40 Total and CCI-40 Memory Scores were associated with Aβ40 in the MCI group, whereas their respective CCI-20 counterparts were not. Furthermore, the CCI-40 Total was significantly associated with the Aβ42 / Aβ40 ratio in NH-B while the CCI-20 was not. Findings for the CCI-12 Total Score was identical to the CCI-20 Memory Total Score. An additional sensitivity analysis excluding days in which participants completed the DMLC more than once yielded largely unchanged results (Supplement Table 2.1–2.3). Furthermore, another sensitivity analysis excluding participants who completed fewer than eight assessments during the 14-day sampling period (*N* = 223) produced results generally consistent with those in the main manuscript albeit with larger confidence intervals (Supplement Table 3.1–3.3).

## Discussion

This study compared traditional self-report measures of subjective cognitive concerns (SCC) with Ecological Momentary Assessment (EMA)-based SCC measures as indicators of blood-based AD biomarker levels in a demographically diverse sample of older adults. We also examined these associations among individuals with and without mild cognitive impairment (MCI), and among NH-Whites and NH-Blacks. Our findings in the entire sample demonstrate that EMA-reported SCC are associated with p-tau181, whereas these relationships were not observed with traditional in-person SCC measures. These results suggest that EMA measures of SCC may help identify individuals with elevated blood-based AD biomarker levels, indicating an increased risk for future cognitive impairment.

The EMA approach (the Daily Memory Lapses Checklist [DMLC]) and a widely used traditional SCC measure (the Cognitive Chane Index [CCI]) may appear to capture similar information about cognitive concerns (DMLC Total Score correlates with Total CCI-20, ρ = 0.52, *p* < 0.001). However, in the fully adjusted models, we did not find an association between traditional self-reported SCC measures and p-tau181, similar to prior studies using traditional SCC approachs [47]. Yet, EMA-reported SCC were associated with p-tau181. The stronger associations between EMA measurement of SCC and biomarkers may stem from the superior ecological validity and real-time assessment of cognitive lapses, rather than retrospective recall. This may reduce the measurement error in traditional approaches and enhance the ability to detect significant associations. Traditional self-report measures often require participants to recall experiences over extended periods, such as the past year, the past 5 years (i.e., CCI timeframe), or even 10 years. These approaches may lead to biased reports, especially among individuals with diminished memory and other cognitive abilities. Daily EMA measures may be important for accurately assessing the frequency (and possibly the impact) of memory and other cognitive problems on daily life and potentially for improving the sensitivity of self-reported cognition to cognitive decline and neuropathology. We propose that EMA may more readily detect subtle markers of cognitive dysfunction [20, 65, 66], providing a dynamic and precise measure of cognitive fluctuations in an individual’s natural environment, potentially boosting the association of SCC with AD-specific biomarkers [67]. 

The utility of EMA SCC measures may lie in their ability to capture risk in early disease stages, given that SCC reported via EMA, not the in-person traditional measures, was associated with p-tau181 in neuropsychologically unimpaired participants. Moreover, SCCs reported via EMA were a particularly important signal for blood-based biomarkers among these unimpaired individuals. We hypothesize that SCC captured via EMA are sensitive to p-tau181 as SCC may signal the early stages of disruption in hippocampal function that are specific to p-tau181. Though Aβ pathology is thought to precede tau pathology, Aβ pathology is relatively common in cognitively normal and cognitively stable older adults. Tau accumulation begins later, closer to the onset of measurable subjective or objective cognitive decline [68–70]. As such, SCC reported via EMA may improve the predictive validity of SCC in anticipating future disease risk, facilitating enrollment into clinical trials and early therapeutic interventions for those most in need, which requires longitudinal testing. This is especially relevant given the increasing emphasis on preventative strategies in neurodegenerative diseases, aimed at intervening before significant neural damage occurs.

As we move along the AD continuum, our results show that SCCs measured by either EMA or CCI were correlated with Aβ (plasma Aβ40, Aβ42/Aβ40 ratio) in the MCI sample, but not in the CU group. This underscores that SCC remain correlated with markers of AD pathology regardless of neuropsychological status. Paper-and-pencil SCC metrics may become useful once objective cognitive impairment is also already detectable. In contrast, SCC captured via EMA may be more sensitive to detection of persons with elevated blood-based AD biomarkers regardless of objective cognitive impairment.

Issues surrounding accessibility, inclusion, and equity should be front and center in aging research and clinical care given the noted health disparities in this realm [71–73]. Ethnic racial minority groups express SCC at higher rates, are at double the risk of dementia misdiagnosis [73], and receive unequal access to dementia care [74]. Our results showed that SCC were associated with p-tau181 and Aβ42/Aβ40 across ethnic/racial groups. However, our analyses suggest that the link between EMA SCC and blood-based biomarkers may be stronger in non-Hispanic White participants, while traditional SCC metrics may be more associated with blood-based biomarkers in non-Hispanic Black participants (although our sensitivity analyses did link EMA SCC with Aβ42/Aβ40 in non-Hispanic Black individuals). These findings should be replicated across larger and more demographically diverse samples to better inform the SCC modality that is most helpful for use in under-represented, minoritized groups. A possibility to be explored in future research is that EMA and traditional SCC could be integrated to provide a more reliable and clinically meaningful assessment for use in diverse populations, with implications for more equitable access to early dementia intervention for minoritized older individuals. Moreover, rural and remote living communities contain a greater proportion of the aging population relative to urban areas [75], while facing outsized barriers to dementia diagnosis and care [76]. In such settings, use of EMA for daily SCC measurements may represent an improvement over traditional methods for SCC assessment, as it is often more accessible and convenient, reducing barriers to access and facilitating follow-up. From the perspective of aging study implementation, we noted no differences in completion rates between the ethnic/racial which bolsters the feasibility of utilizing EMA in multi-ethnic populations. However, MCI groups had lower completion rates than CU. Therefore, additional consideration must be given when using EMA in MCI participants as a prompted rather than self-initiated design may be more effective.

Despite the promise of novel EMA tools, traditional measures of SCC play a critical role in research and clinical work and remain the gold standard for use in aging populations. Traditional paper-and-pencil, in person SCC measures are brief, affordable, and can be completed in one sitting [53]. Although widespread, smartphone use in older adulthood is not ubiquitous, meaning not every at-risk individual would have access to EMA approaches. Given that we collected data by EMA only once daily via smartphone, it is possible that future studies may employ web-based methods implemented through tablets and/or personal computers that may be suitable for collection of DMLC data in clinical practice. Future research that investigates a range of complementary and equivalent technology-based approaches to SCC assessment (e.g., smartphone, tablet, and personal computer) may improve accessibility and convenience for older adults, researchers, and clinicians alike. Regarding paper-and-pencil SCC measures, our study demonstrated associations between non-Memory CCI items and Aβ in our sample, and between CCI memory and non-memory items and Aβ in participants with MCI. These results not only underscore the utility of traditional SCC tools but also point out the importance of comprehensively assessing subjective concerns across broad cognitive domains rather solely focusing only memory changes. Indeed, concerns related to memory changes are incredibly widespread in aging populations, thus merely emphasizing memory concerns may not be sensitive or specific to individuals who may be at the highest risk [11, 77]. Furthermore, discrepancies based on the version of the CCI (CCI-20 or CCI-40) used indicate that the optimal number of items and content remain unresolved, underscoring the need for further research.

Limitations of our study include: (1) reliance solely on self-reported SCC with no informant report; (2) reliance on self-initiated reports rather than prompted reports; (3) EMA SCC may be more burdensome than traditional single-shot pen-and-paper tests as it requires successive testing over several days, (4) we assessed only cross-sectional associations between biomarkers and SCCs, and thus further work needs to be performed on whether the DMLC predicts incident MCI in order to use DMLC as a screener. (5) our findings did not include people of Hispanic or other backgrounds limiting generalizability, and 7) we have a limited sample size, specially within our stratified analysis. Future directions include: (1) utilizing machine learning and advanced analytical techniques to integrate traditional and EMA approaches, enhancing the sensitivity of SCCs to AD biomarkers; (2) harnessing the extensive data captured through intensive repeated measures of SCC via EMA; (3) integrate informant reports—as suggested by prior work using instruments such as the ECog [47] to address the challenges posed by anosognosia in later stages of the disease and (4) Future work should examine the predictive validity of the DMLC, blood-based biomarkers, and their combination for incident cognitive impairment, MCI and dementia.

Overall, our work demonstrates that using EMA approaches to measuring SCC enhances correlations with key AD biomarkers. Ultimately, EMA-based approaches to SCC may be used to identify individuals at high risk for AD who require further assessment with blood-based biomarkers. Perhaps EMA measures of SCC will serve as an effective adjunct or proxy for monitoring AD progression and evaluating pathology in routine clinical settings across diverse populations. EMA measurements of SCC may be superior for detecting risk in the earliest preclinical stages, suitable for use with demographically diverse patient populations, including those in remote or rural settings, thus highlighting its potential for broader applicability and inclusivity. The development of tablet and personal computer screens may increase the applicability of this approach. In conclusion, our findings provide novel insights into detecting early cognitive decline through SCC, significantly enriching AD research. This work lays a vital foundation for future research and clinical practices aimed at reducing the burden of ADRD utilizing novel, remotely administer digital tools.

## Electronic Supplementary Material

Below is the link to the electronic supplementary material.

Supplementary Material 1

## Data Availability

The dataset supporting the conclusion of this article is available by request from corresponding author.
