# Supplementary material for: Enhanced associations between subjective cognitive concerns and blood-based AD biomarkers using a novel EMA approach
Source: Alzheimers Res Ther. 2025 Apr 15;17:82. doi: 10.1186/s13195-025-01720-y (PMC11998261; doi:10.1186/s13195-025-01720-y)
Supplement: Supplementary file 1 — Supplementary Material 1 [file 13195_2025_1720_MOESM1_ESM.docx]

**Supplement**

Supplemental Table 1: Daily Memory Lapses Checklist (DMLC)

- **DMLC Memory**
  - During the day today did you forget any of the following? <Check all that apply>
  - An errand/chore
  - To take medicine or take medicine on time
  - To attend a planned meeting or event (e.g., appointment, social event)
  - To make a call
  - Why you entered a room
  - To bring something with you
  - None
- **DMLC Memory**
  - Did you forget any of these other things during the day today? <Check all that apply>
  - Someone’s name
  - Where something was placed
  - A word during conversation
  - Something you wanted to recall
  - Other: open-ended
  - None
- **DMLC Non-Memory**
  - During the day today did you have any problems with: <Check all that apply>
  - Keeping track of what you were doing
  - Being organized
  - Making decisions
  - Thinking quickly
  - Thinking clearly
  - Finding your way around
  - Completing a task
  - None

Supplemental Table 2: Cognitive Change Index (20 Item Version)

Please rate your current level of ability compared to 5 years ago and the severity of any current problems.

| **Compared to five years ago ...** | **Normal Ability**  ***(No Change)*** | **Slight/Occasional Problem**  ***(Minimal Change)*** | **Mild Problem**  ***(Some Change)*** | **Moderate Problem**  ***(Clearly Noticeable Change)*** | **Severe Problem**  ***(Much Worse)*** |
| --- | --- | --- | --- | --- | --- |
| 1. Recalling information when I really try | 1 | 2 | 3 | 4 | 5 |
| 2. Remembering names and faces of new people I meet | 1 | 2 | 3 | 4 | 5 |
| 3. Remembering things that have happened recently | 1 | 2 | 3 | 4 | 5 |
| 4. Recalling conversations a few days later | 1 | 2 | 3 | 4 | 5 |
| 5. Remembering where things are usually kept | 1 | 2 | 3 | 4 | 5 |
| 6. Remembering new information told to me | 1 | 2 | 3 | 4 | 5 |
| 7. Remembering where I placed familiar objects | 1 | 2 | 3 | 4 | 5 |
| 8. Remembering what I intended to do | 1 | 2 | 3 | 4 | 5 |
| 9. Remembering names of family members and friends | 1 | 2 | 3 | 4 | 5 |
| 10. Remembering without notes and reminders | 1 | 2 | 3 | 4 | 5 |
| 11. People who know me would find that my memory is | 1 | 2 | 3 | 4 | 5 |
| 12. Remembering things compared to my age group | 1 | 2 | 3 | 4 | 5 |
| 13. Making decisions about everyday matters | 1 | 2 | 3 | 4 | 5 |
| 14. Reasoning through a complicated problem | 1 | 2 | 3 | 4 | 5 |
| 15. Focusing on goals and carrying out a plan | 1 | 2 | 3 | 4 | 5 |
| 16. Shifting easily from one activity to the next | 1 | 2 | 3 | 4 | 5 |
| 17. Organizing my daily activities | 1 | 2 | 3 | 4 | 5 |
| 18. Understanding conversations | 1 | 2 | 3 | 4 | 5 |
| 19. Expressing myself when speaking | 1 | 2 | 3 | 4 | 5 |
| 20. Following a story in a book, movie, or TV | 1 | 2 | 3 | 4 | 5 |

1. **Sensitivity Analysis 1: Comparing CCI-20 to CCI-40**

Results using the CCI-20 and CCI-40 are largely similar, with some notable differences in their associations with the Aβ42/Aβ40 ratio. We found that the CCI-20 Non-Memory items are associated with the Aβ42/Aβ40 ratio in the entire sample, whereas the CCI-40 Non-Memory items are not. The CCI-20 Total score is not associated with the Aβ42/Aβ40 ratio in the NH-B group, but the CCI-40 Total score is significantly associated (β = -0.19, p = 0.037). Lastly, the CCI-20 Total are not associated with Aβ40 in the MCI group, nor are the CCI-20 Memory Items, while both the CCI-40 Total (β = 0.22, p = 0.029). and the CCI-40 Memory items show a significant association (β = 0.20, p = 0.042).

Supplemental Table 1.1: Associations between CCI-40 and Biomarker on the entire sample (N = 254), adjusted for the following covariates: age, gender, race/ethnicity, cognitive status (MCI/CU), years of education, and depression (as measured by the Geriatric Depression Scale). Bold indicates significant association at $\alpha=0.05$

| **SCC Metric** | Aβ40 | | Aβ42 | | GFAP | | NfL | | p-tau181 | | Aβ42 / Aβ40 | |
| --- | --- | --- | --- | --- | --- | --- | --- | --- | --- | --- | --- | --- |
|  | **Beta**  **(95% CI)***^1^* | **p-value** | **Beta**  **(95% CI)***^1^* | **p-value** | **Beta**  **(95% CI)***^1^* | **p-value** | **Beta**  **(95% CI)***^1^* | **p-value** | **Beta**  **(95% CI)***^1^* | **p-value** | **Beta**  **(95% CI)***^1^* | **p-value** |
| CCI-40 Total | 0.03  (-0.09 to 0.14) | 0.658 | -0.04  (-0.16 to 0.08) | 0.54 | -0.02  (-0.16 to 0.11) | 0.754 | 0.05  (-0.05 to 0.15) | 0.328 | 0.12  (-0.01 to 0.26) | 0.073 | -0.11  (-0.23 to 0.02) | 0.088 |
| CCI-40 Memory | 0.01  (-0.10 to 0.12) | 0.881 | -0.05  (-0.17 to 0.07) | 0.403 | -0.04  (-0.17 to 0.09) | 0.561 | 0.03  (-0.07 to 0.13) | 0.569 | 0.12  (-0.02 to 0.25) | 0.083 | -0.09  (-0.21 to 0.03) | 0.154 |
| CCI-40 Not Memory | 0.04  (-0.07 to 0.16) | 0.459 | -0.02  (-0.14 to 0.11) | 0.796 | 0.00  (-0.13 to 0.14) | 0.956 | 0.07  (-0.03 to 0.17) | 0.179 | 0.11  (-0.02 to 0.24) | 0.106 | -0.11  (-0.24 to 0.01) | 0.07 |
| *^1^* CI = Confidence Interval | | | | | | | | | | | | |

Supplementary Table 1.2: Associations between each SCC Metric and Biomarker stratified by Cognitive Status (MCI / CU), adjusted for the following covariates: age, gender, race/ethnicity, years of education, and depression (as measured by the Geriatric Depression Scale). Bold indicates significant association at $\alpha=0.05$

| **Mild Cognitive Impairment (N = 75)** | | | | | | | | | | | | |
| --- | --- | --- | --- | --- | --- | --- | --- | --- | --- | --- | --- | --- |
| **SCC Metric** | Aβ40 | | Aβ42 | | GFAP | | NfL | | p-tau181 | | Aβ42 / Aβ40 | |
|  | **Beta**  **(95% CI)***^1^* | **p-value** | **Beta**  **(95% CI)***^1^* | **p-value** | **Beta**  **(95% CI)***^1^* | **p-value** | **Beta**  **(95% CI)***^1^* | **p-value** | **Beta**  **(95% CI)***^1^* | **p-value** | **Beta**  **(95% CI)***^1^* | **p-value** |
| CCI-40 Total | 0.22  (0.02 to 0.41) | **0.029** | -0.03  (-0.25 to 0.20) | 0.822 | -0.09  (-0.33 to 0.16) | 0.484 | 0.06  (-0.07 to 0.20) | 0.347 | 0.21  (-0.03 to 0.46) | 0.087 | -0.39  (-0.62 to -0.15) | **0.002** |
| CCI-40 Memory | 0.20  (0.01 to 0.40) | **0.042** | -0.05  (-0.27 to 0.18) | 0.691 | -0.11  (-0.35 to 0.14) | 0.386 | 0.03  (-0.10 to 0.16) | 0.654 | 0.19  (-0.05 to 0.44) | 0.119 | -0.38  (-0.61 to -0.14) | **0.002** |
| CCI-40 Not Memory | 0.21  (0.02 to 0.40) | **0.03** | 0.00  (-0.22 to 0.23) | 0.995 | -0.05  (-0.30 to 0.19) | 0.668 | 0.10  (-0.03 to 0.23) | 0.145 | 0.22  (-0.03 to 0.46) | 0.083 | -0.37  (-0.60 to -0.13) | **0.003** |
| **Cognitively Unimpaired (N = 179)** | | | | | | | | | | | | |
| **SCC Metric** | Aβ40 | | Aβ42 | | GFAP | | NfL | | p-tau181 | | Aβ42 / Aβ40 | |
|  | **Beta**  **(95% CI)***^1^* | **p-value** | **Beta**  **(95% CI)***^1^* | **p-value** | **Beta**  **(95% CI)***^1^* | **p-value** | **Beta**  **(95% CI)***^1^* | **p-value** | **Beta**  **(95% CI)***^1^* | **p-value** | **Beta**  **(95% CI)***^1^* | **p-value** |
| CCI-40 Total | -0.08  (-0.22 to 0.05) | 0.231 | -0.04  (-0.19 to 0.10) | 0.553 | 0.01  (-0.16 to 0.17) | 0.918 | 0.04  (-0.10 to 0.18) | 0.563 | 0.04  (-0.12 to 0.21) | 0.598 | 0.06  (-0.08 to 0.21) | 0.388 |
| CCI-40 Memory | -0.09  (-0.23 to 0.05) | 0.198 | -0.04  (-0.19 to 0.10) | 0.552 | 0.00  (-0.16 to 0.16) | 0.996 | 0.03  (-0.11 to 0.16) | 0.694 | 0.06  (-0.10 to 0.22) | 0.457 | 0.08  (-0.06 to 0.22) | 0.287 |
| CCI-40 Not Memory | -0.06  (-0.20 to 0.08) | 0.367 | -0.04  (-0.18 to 0.11) | 0.616 | 0.02  (-0.15 to 0.18) | 0.828 | 0.05  (-0.09 to 0.18) | 0.476 | 0.02  (-0.15 to 0.18) | 0.855 | 0.04  (-0.11 to 0.18) | 0.627 |
| *^1^* CI = Confidence Interval | | | | | | | | | | | | |

Supplementary Table 1.3: Associations between each SCC Metric and Biomarker stratified by Race / Ethnicity (NH-White & NH-Black) adjusted for the following covariates: age, gender, cognitive status (MCI / CU), years of education, and depression (as measured by the Geriatric Depression Scale). Bold indicates significant association at $\alpha=0.05$

| **Non-Hispanic Black (N = 103)** | | | | | | | | | | | | |
| --- | --- | --- | --- | --- | --- | --- | --- | --- | --- | --- | --- | --- |
| **SCC Metric** | Aβ40 | | Aβ42 | | GFAP | | NfL | | p-tau181 | | Aβ42 / Aβ40 | |
|  | **Beta**  **(95% CI)***^1^* | **p-value** | **Beta**  **(95% CI)***^1^* | **p-value** | **Beta**  **(95% CI)***^1^* | **p-value** | **Beta**  **(95% CI)***^1^* | **p-value** | **Beta**  **(95% CI)***^1^* | **p-value** | **Beta**  **(95% CI)***^1^* | **p-value** |
| CCI-40 Total | 0.06  (-0.10 to 0.23) | 0.431 | -0.06  (-0.24 to 0.11) | 0.487 | 0.02  (-0.18 to 0.23) | 0.82 | 0.02  (-0.13 to 0.16) | 0.835 | 0.19  (0.00 to 0.38) | **0.046** | -0.19  (-0.37 to -0.01) | **0.037** |
| CCI-40 Memory | 0.02  (-0.14 to 0.18) | 0.814 | -0.11  (-0.28 to 0.06) | 0.214 | 0.00  (-0.21 to 0.20) | 0.97 | -0.02  (-0.17 to 0.12) | 0.755 | 0.20  (0.02 to 0.38) | **0.03** | -0.18  (-0.36 to 0.00) | 0.053 |
| CCI-40 Not Memory | 0.11  (-0.05 to 0.27) | 0.176 | 0.00  (-0.17 to 0.18) | 0.96 | 0.05  (-0.15 to 0.26) | 0.604 | 0.06  (-0.09 to 0.21) | 0.419 | 0.15  (-0.04 to 0.33) | 0.126 | -0.18  (-0.37 to 0.00) | **0.048** |
| **Non-Hispanic White (N = 124)** | | | | | | | | | | | | |
| **SCC Metric** | Aβ40 | | Aβ42 | | GFAP | | NfL | | p-tau181 | | Aβ42 / Aβ40 | |
|  | **Beta**  **(95% CI)***^1^* | **p-value** | **Beta**  **(95% CI)***^1^* | **p-value** | **Beta**  **(95% CI)***^1^* | **p-value** | **Beta**  **(95% CI)***^1^* | **p-value** | **Beta**  **(95% CI)***^1^* | **p-value** | **Beta**  **(95% CI)***^1^* | **p-value** |
| CCI-40 Total | -0.02  (-0.19 to 0.15) | 0.819 | 0.01  (-0.17 to 0.19) | 0.903 | -0.07  (-0.27 to 0.14) | 0.508 | 0.05  (-0.11 to 0.21) | 0.512 | 0.02  (-0.19 to 0.23) | 0.854 | 0.07  (-0.11 to 0.25) | 0.449 |
| CCI-40 Memory | -0.02  (-0.19 to 0.15) | 0.826 | 0.01  (-0.17 to 0.19) | 0.892 | -0.08  (-0.28 to 0.12) | 0.444 | 0.04  (-0.12 to 0.20) | 0.629 | -0.01  (-0.22 to 0.20) | 0.939 | 0.07  (-0.11 to 0.25) | 0.454 |
| CCI-40 Not Memory | -0.02  (-0.19 to 0.16) | 0.842 | 0.01  (-0.17 to 0.18) | 0.933 | -0.04  (-0.25 to 0.16) | 0.671 | 0.06  (-0.10 to 0.22) | 0.452 | 0.05  (-0.16 to 0.26) | 0.642 | 0.06  (-0.12 to 0.24) | 0.524 |
| *^1^* CI = Confidence Interval | | | | | | | | | | | | |

**Sensitivity Analysis 2: Excluding Data when Participants Filled out the DMLC Multiple Times during the Same Day**

Results excluding data for days in which participants completed the DMLC more than once results in the exclusion of one additional participant. Results remain largely the same. The exceptions are: (1) DMLC Memory Total is associated with Aβ42/Aβ40 in NH-Blacks and with p-tau181 in NH-Whites, whereas it is not in our main analysis; and (2) CCI-20 Total Score is associated with Aβ40 in the MCI group, whereas it is not in our main analysis.

Supplemental Table 2.1: Associations between each SCC Metric and Biomarker on the entire sample (N = 253), adjusted for the following covariates: age, gender, race/ethnicity, cognitive status (MCI/CU), years of education, and depression (as measured by the Geriatric Depression Scale). Bold indicates significant association at $\alpha=0.05$

| **SCC Metric** | Aβ40 | | Aβ42 | | GFAP | | NfL | | p-tau181 | | Aβ42 / Aβ40 | |
| --- | --- | --- | --- | --- | --- | --- | --- | --- | --- | --- | --- | --- |
|  | **Beta**  **(95% CI)***^1^* | **p-value** | **Beta**  **(95% CI)***^1^* | **p-value** | **Beta**  **(95% CI)***^1^* | **p-value** | **Beta**  **(95% CI)***^1^* | **p-value** | **Beta**  **(95% CI)***^1^* | **p-value** | **Beta**  **(95% CI)***^1^* | **p-value** |
| DMLC Total | 0.07  (-0.04 to 0.17) | 0.223 | 0.00  (-0.11 to 0.12) | 0.984 | -0.01  (-0.14 to 0.12) | 0.851 | 0.03  (-0.06 to 0.13) | 0.519 | 0.21  (0.08 to 0.33) | **0.001** | -0.11  (-0.22 to 0.01) | 0.075 |
| DMLC Memory | 0.09  (-0.02 to 0.19) | 0.119 | 0.03  (-0.09 to 0.14) | 0.633 | 0.00  (-0.13 to 0.13) | 0.97 | 0.07  (-0.03 to 0.16) | 0.182 | 0.23  (0.10 to 0.35) | **<0.001** | -0.09  (-0.20 to 0.03) | 0.142 |
| DMLC Not-Memory | 0.03  (-0.08 to 0.14) | 0.59 | -0.03  (-0.15 to 0.08) | 0.549 | -0.03  (-0.16 to 0.10) | 0.641 | -0.02  (-0.12 to 0.08) | 0.679 | 0.14  (0.02 to 0.27) | **0.028** | -0.11  (-0.23 to 0.00) | 0.06 |
| CCI-20 Total | 0.00  (-0.11 to 0.12) | 0.953 | -0.08  (-0.20 to 0.04) | 0.193 | -0.02  (-0.16 to 0.12) | 0.773 | 0.03  (-0.07 to 0.13) | 0.559 | 0.13  (-0.01 to 0.26) | 0.064 | -0.12  (-0.25 to 0.00) | 0.052 |
| CCI-20 Memory | -0.02  (-0.13 to 0.09) | 0.734 | -0.10  (-0.22 to 0.03) | 0.12 | -0.03  (-0.17 to 0.10) | 0.632 | 0.02  (-0.08 to 0.12) | 0.708 | 0.12  (-0.01 to 0.26) | 0.071 | -0.09  (-0.22 to 0.03) | 0.128 |
| CCI-20 Not Memory | 0.05  (-0.07 to 0.16) | 0.422 | -0.03  (-0.16 to 0.09) | 0.611 | 0.01  (-0.13 to 0.15) | 0.885 | 0.04  (-0.06 to 0.15) | 0.402 | 0.10  (-0.03 to 0.24) | 0.136 | -0.14  (-0.27 to -0.02) | **0.024** |
| *^1^* CI = Confidence Interval | | | | | | | | | | | | |

Supplemental Table 2.2: Associations between each SCC Metric and Biomarker stratified by Cognitive Status (MCI / CU), adjusted for the following covariates: age, gender, race/ethnicity, years of education, and depression (as measured by the Geriatric Depression Scale). Bold indicates significant association at $\alpha=0.05$

| **Mild Cognitive Impairment (N = 74)** | | | | | | | | | | | | |
| --- | --- | --- | --- | --- | --- | --- | --- | --- | --- | --- | --- | --- |
| **SCC Metric** | Aβ40 | | Aβ42 | | GFAP | | NfL | | p-tau181 | | Aβ42 / Aβ40 | |
|  | **Beta**  **(95% CI)***^1^* | **p-value** | **Beta**  **(95% CI)***^1^* | **p-value** | **Beta**  **(95% CI)***^1^* | **p-value** | **Beta**  **(95% CI)***^1^* | **p-value** | **Beta**  **(95% CI)***^1^* | **p-value** | **Beta**  **(95% CI)***^1^* | **p-value** |
| DMLC Total | 0.31  (0.13 to 0.49) | **0.001** | 0.10  (-0.12 to 0.31) | 0.383 | 0.14  (-0.10 to 0.38) | 0.258 | 0.10  (-0.03 to 0.23) | 0.14 | 0.23  (-0.01 to 0.46) | 0.063 | -0.33  (-0.56 to -0.09) | **0.007** |
| DMLC Memory | 0.32  (0.14 to 0.50) | **<0.001** | 0.12  (-0.10 to 0.34) | 0.268 | 0.09  (-0.15 to 0.33) | 0.48 | 0.09  (-0.04 to 0.22) | 0.195 | 0.18  (-0.06 to 0.42) | 0.132 | -0.29  (-0.53 to -0.06) | **0.016** |
| DMLC Not-Memory | 0.26  (0.07 to 0.44) | **0.007** | 0.05  (-0.17 to 0.26) | 0.679 | 0.20  (-0.03 to 0.44) | 0.089 | 0.11  (-0.02 to 0.23) | 0.104 | 0.27  (0.04 to 0.51) | **0.025** | -0.35  (-0.58 to -0.11) | **0.004** |
| CCI-20 Total | 0.20  (0.00 to 0.40) | **0.049** | -0.07  (-0.30 to 0.16) | 0.54 | -0.08  (-0.33 to 0.17) | 0.518 | 0.05  (-0.09 to 0.19) | 0.464 | 0.23  (-0.02 to 0.48) | 0.066 | -0.41  (-0.65 to -0.17) | **0.001** |
| CCI-20 Memory | 0.17  (-0.03 to 0.37) | 0.094 | -0.10  (-0.33 to 0.13) | 0.381 | -0.09  (-0.34 to 0.16) | 0.458 | 0.02  (-0.11 to 0.16) | 0.729 | 0.21  (-0.04 to 0.46) | 0.092 | -0.38  (-0.62 to -0.14) | **0.002** |
| CCI-20 Not Memory | 0.21  (0.02 to 0.41) | **0.033** | 0.00  (-0.23 to 0.23) | 0.985 | -0.04  (-0.30 to 0.21) | 0.725 | 0.09  (-0.05 to 0.23) | 0.192 | 0.22  (-0.02 to 0.47) | 0.077 | -0.37  (-0.62 to -0.13) | **0.003** |
| **Cognitively Unimpaired (N = 179)** | | | | | | | | | | | | |
| **SCC Metric** | Aβ40 | | Aβ42 | | GFAP | | NfL | | p-tau181 | | Aβ42 / Aβ40 | |
|  | **Beta**  **(95% CI)***^1^* | **p-value** | **Beta**  **(95% CI)***^1^* | **p-value** | **Beta**  **(95% CI)***^1^* | **p-value** | **Beta**  **(95% CI)***^1^* | **p-value** | **Beta**  **(95% CI)***^1^* | **p-value** | **Beta**  **(95% CI)***^1^* | **p-value** |
| DMLC Total | -0.05  (-0.18 to 0.08) | 0.442 | -0.04  (-0.18 to 0.10) | 0.565 | -0.09  (-0.24 to 0.06) | 0.243 | 0.00  (-0.13 to 0.12) | 0.973 | 0.19  (0.04 to 0.34) | **0.012** | 0.02  (-0.12 to 0.15) | 0.818 |
| DMLC Memory | -0.04  (-0.17 to 0.09) | 0.587 | -0.02  (-0.15 to 0.12) | 0.824 | -0.05  (-0.20 to 0.10) | 0.524 | 0.06  (-0.07 to 0.18) | 0.39 | 0.24  (0.09 to 0.39) | **0.001** | 0.03  (-0.10 to 0.17) | 0.614 |
| DMLC Not-Memory | -0.06  (-0.19 to 0.07) | 0.382 | -0.06  (-0.20 to 0.07) | 0.378 | -0.12  (-0.27 to 0.03) | 0.118 | -0.07  (-0.20 to 0.05) | 0.257 | 0.09  (-0.06 to 0.24) | 0.249 | -0.01  (-0.14 to 0.12) | 0.872 |
| CCI-20 Total | -0.10  (-0.24 to 0.04) | 0.145 | -0.09  (-0.23 to 0.06) | 0.249 | 0.01  (-0.15 to 0.18) | 0.862 | 0.02  (-0.12 to 0.16) | 0.773 | 0.05  (-0.11 to 0.21) | 0.551 | 0.04  (-0.10 to 0.18) | 0.592 |
| CCI-20 Memory | -0.11  (-0.24 to 0.03) | 0.132 | -0.08  (-0.22 to 0.07) | 0.288 | 0.00  (-0.16 to 0.16) | 0.995 | 0.02  (-0.12 to 0.15) | 0.791 | 0.06  (-0.10 to 0.22) | 0.434 | 0.06  (-0.08 to 0.20) | 0.403 |
| CCI-20 Not Memory | -0.07  (-0.21 to 0.07) | 0.334 | -0.08  (-0.23 to 0.07) | 0.31 | 0.04  (-0.13 to 0.21) | 0.647 | 0.02  (-0.12 to 0.16) | 0.8 | 0.01  (-0.16 to 0.17) | 0.944 | -0.01  (-0.16 to 0.13) | 0.842 |
| *^1^* CI = Confidence Interval | | | | | | | | | | | | |

Supplemental Table 2.3: Associations between each SCC Metric and Biomarker stratified by Race / Ethnicity (NH-White & NH-Black) adjusted for the following covariates: age, gender, cognitive status (MCI / CU), years of education, and depression (as measured by the Geriatric Depression Scale). Bold indicates significant association at $\alpha=0.05$

| **Non-Hispanic Black (N = 103)** | | | | | | | | | | | | |
| --- | --- | --- | --- | --- | --- | --- | --- | --- | --- | --- | --- | --- |
| **SCC Metric** | Aβ40 | | Aβ42 | | GFAP | | NfL | | p-tau181 | | Aβ42 / Aβ40 | |
|  | **Beta**  **(95% CI)***^1^* | **p-value** | **Beta**  **(95% CI)***^1^* | **p-value** | **Beta**  **(95% CI)***^1^* | **p-value** | **Beta**  **(95% CI)***^1^* | **p-value** | **Beta**  **(95% CI)***^1^* | **p-value** | **Beta**  **(95% CI)***^1^* | **p-value** |
| DMLC Total | 0.06  (-0.10 to 0.22) | 0.444 | -0.03  (-0.20 to 0.14) | 0.702 | 0.11  (-0.09 to 0.31) | 0.27 | 0.02  (-0.12 to 0.16) | 0.762 | 0.15  (-0.03 to 0.33) | 0.098 | -0.17  (-0.34 to 0.01) | 0.064 |
| DMLC Memory | 0.07  (-0.09 to 0.22) | 0.401 | -0.01  (-0.18 to 0.16) | 0.892 | 0.11  (-0.09 to 0.31) | 0.261 | 0.01  (-0.14 to 0.15) | 0.924 | 0.17  (-0.02 to 0.35) | 0.073 | -0.14  (-0.32 to 0.04) | 0.122 |
| DMLC Not-Memory | 0.04  (-0.11 to 0.20) | 0.572 | -0.06  (-0.23 to 0.11) | 0.503 | 0.09  (-0.11 to 0.29) | 0.367 | 0.04  (-0.10 to 0.18) | 0.573 | 0.11  (-0.07 to 0.29) | 0.229 | -0.18  (-0.35 to -0.01) | **0.043** |
| CCI-20 Total | 0.03  (-0.13 to 0.19) | 0.748 | -0.11  (-0.28 to 0.07) | 0.228 | 0.03  (-0.17 to 0.24) | 0.746 | -0.01  (-0.15 to 0.14) | 0.938 | 0.19  (0.01 to 0.38) | **0.04** | -0.18  (-0.36 to 0.00) | 0.052 |
| CCI-20 Memory | -0.02  (-0.18 to 0.14) | 0.81 | -0.14  (-0.31 to 0.03) | 0.101 | 0.02  (-0.18 to 0.23) | 0.812 | -0.04  (-0.18 to 0.11) | 0.605 | 0.20  (0.02 to 0.38) | **0.032** | -0.15  (-0.33 to 0.03) | 0.105 |
| CCI-20 Not Memory | 0.11  (-0.05 to 0.27) | 0.188 | -0.01  (-0.19 to 0.17) | 0.9 | 0.04  (-0.16 to 0.25) | 0.678 | 0.06  (-0.09 to 0.21) | 0.442 | 0.14  (-0.05 to 0.32) | 0.159 | -0.20  (-0.38 to -0.02) | **0.034** |
| **Non-Hispanic White (N = 123)** | | | | | | | | | | | | |
| **SCC Metric** | Aβ40 | | Aβ42 | | GFAP | | NfL | | p-tau181 | | Aβ42 / Aβ40 | |
|  | **Beta**  **(95% CI)***^1^* | **p-value** | **Beta**  **(95% CI)***^1^* | **p-value** | **Beta**  **(95% CI)***^1^* | **p-value** | **Beta**  **(95% CI)***^1^* | **p-value** | **Beta**  **(95% CI)***^1^* | **p-value** | **Beta**  **(95% CI)***^1^* | **p-value** |
| DMLC Total | 0.12  (-0.05 to 0.29) | 0.162 | 0.05  (-0.13 to 0.22) | 0.585 | -0.08  (-0.28 to 0.12) | 0.431 | 0.03  (-0.13 to 0.19) | 0.692 | 0.31  (0.11 to 0.51) | **0.002** | -0.11  (-0.29 to 0.07) | 0.229 |
| DMLC Memory | 0.16  (-0.01 to 0.33) | 0.067 | 0.09  (-0.09 to 0.26) | 0.321 | -0.04  (-0.25 to 0.16) | 0.669 | 0.11  (-0.05 to 0.27) | 0.175 | 0.34  (0.14 to 0.54) | **<0.001** | -0.10  (-0.29 to 0.08) | 0.265 |
| DMLC Not-Memory | 0.05  (-0.12 to 0.22) | 0.588 | -0.01  (-0.19 to 0.16) | 0.889 | -0.11  (-0.31 to 0.09) | 0.289 | -0.07  (-0.23 to 0.09) | 0.367 | 0.20  (0.00 to 0.40) | **0.047** | -0.09  (-0.27 to 0.09) | 0.3 |
| CCI-20 Total | -0.04  (-0.22 to 0.13) | 0.645 | -0.05  (-0.23 to 0.13) | 0.591 | -0.10  (-0.31 to 0.10) | 0.333 | 0.03  (-0.13 to 0.19) | 0.714 | 0.00  (-0.21 to 0.21) | 0.994 | 0.03  (-0.16 to 0.22) | 0.756 |
| CCI-20 Memory | -0.04  (-0.22 to 0.13) | 0.645 | -0.05  (-0.23 to 0.13) | 0.581 | -0.13  (-0.33 to 0.08) | 0.223 | 0.04  (-0.13 to 0.20) | 0.661 | -0.02  (-0.23 to 0.19) | 0.83 | 0.03  (-0.16 to 0.21) | 0.773 |
| CCI-20 Not Memory | -0.03  (-0.20 to 0.15) | 0.758 | -0.03  (-0.21 to 0.15) | 0.738 | -0.01  (-0.22 to 0.19) | 0.889 | 0.01  (-0.15 to 0.17) | 0.922 | 0.05  (-0.16 to 0.26) | 0.646 | 0.02  (-0.16 to 0.21) | 0.798 |
| *^1^* CI = Confidence Interval | | | | | | | | | | | | |

**Sensitivity Analysis 3: Excluding participants with less than 8 assessments during the 14-day sampling period**

Results excluding participants with less than 8 assessments during the 14-day sampling period (N = 223) Main conclusions hold although some associations are no longer significant given the reduced sample size. In the entire sample, the association between CCI-20 Not Memory is no longer linked to Aβ42/Aβ40. In the MCI group, DMLC remains associated with Aβ40 but is no longer associated with Aβ42/Aβ40. Results in the CU group remain unchanged. The association between CCI and p-tau181 in NH-Blacks is no longer significant, although point estimates remain similar (with a larger confidence interval) while results in NH-Whites remain stable.

Supplemental Table 3.1: Associations between each SCC Metric and Biomarker on the entire sample (N = 223), adjusted for the following covariates: age, gender, race/ethnicity, cognitive status (MCI/CU), years of education, and depression (as measured by the Geriatric Depression Scale). Bold indicates significant association at $\alpha=0.05$

| **SCC Metric** | Aβ40 | | Aβ42 | | GFAP | | NfL | | p-tau181 | | Aβ42 / Aβ40 | |
| --- | --- | --- | --- | --- | --- | --- | --- | --- | --- | --- | --- | --- |
|  | **Beta**  **(95% CI)***^1^* | **p-value** | **Beta**  **(95% CI)***^1^* | **p-value** | **Beta**  **(95% CI)***^1^* | **p-value** | **Beta**  **(95% CI)***^1^* | **p-value** | **Beta**  **(95% CI)***^1^* | **p-value** | **Beta**  **(95% CI)***^1^* | **p-value** |
| DMLC Total | 0.07  (-0.05 to 0.19) | 0.255 | 0.04  (-0.09 to 0.16) | 0.562 | -0.01  (-0.15 to 0.13) | 0.935 | 0.02  (-0.09 to 0.13) | 0.71 | 0.21  (0.08 to 0.34) | **0.002** | -0.06  (-0.18 to 0.07) | 0.366 |
| DMLC Memory | 0.09  (-0.03 to 0.21) | 0.135 | 0.06  (-0.07 to 0.18) | 0.36 | 0.01  (-0.13 to 0.15) | 0.861 | 0.05  (-0.05 to 0.16) | 0.317 | 0.23  (0.10 to 0.36) | **<0.001** | -0.05  (-0.18 to 0.07) | 0.384 |
| DMLC Not-Memory | 0.03  (-0.09 to 0.14) | 0.67 | 0.00  (-0.12 to 0.12) | 0.994 | -0.03  (-0.17 to 0.11) | 0.677 | -0.03  (-0.14 to 0.08) | 0.58 | 0.14  (0.01 to 0.27) | **0.038** | -0.05  (-0.17 to 0.07) | 0.438 |
| CCI-20 Total | -0.01  (-0.14 to 0.11) | 0.834 | -0.05  (-0.19 to 0.08) | 0.424 | -0.04  (-0.19 to 0.11) | 0.621 | 0.01  (-0.10 to 0.13) | 0.833 | 0.10  (-0.04 to 0.24) | 0.166 | -0.05  (-0.19 to 0.08) | 0.435 |
| CCI-20 Memory | -0.03  (-0.15 to 0.09) | 0.635 | -0.06  (-0.19 to 0.07) | 0.36 | -0.04  (-0.19 to 0.10) | 0.553 | 0.01  (-0.11 to 0.12) | 0.888 | 0.11  (-0.03 to 0.25) | 0.118 | -0.02  (-0.15 to 0.11) | 0.726 |
| CCI-20 Not Memory | 0.02  (-0.10 to 0.15) | 0.732 | -0.03  (-0.16 to 0.11) | 0.709 | -0.01  (-0.17 to 0.14) | 0.858 | 0.02  (-0.10 to 0.13) | 0.775 | 0.05  (-0.09 to 0.19) | 0.494 | -0.09  (-0.22 to 0.04) | 0.168 |
| *^1^* CI = Confidence Interval | | | | | | | | | | | | |

Supplemental Table 3.2: Associations between each SCC Metric and Biomarker stratified by Cognitive Status (MCI / CU), adjusted for the following covariates: age, gender, race/ethnicity, years of education, and depression (as measured by the Geriatric Depression Scale). Bold indicates significant association at $\alpha=0.05$

| **Mild Cognitive Impairment (N = 61)** | | | | | | | | | | | | |
| --- | --- | --- | --- | --- | --- | --- | --- | --- | --- | --- | --- | --- |
| **SCC Metric** | Aβ40 | | Aβ42 | | GFAP | | NfL | | p-tau181 | | Aβ42 / Aβ40 | |
|  | **Beta**  **(95% CI)***^1^* | **p-value** | **Beta**  **(95% CI)***^1^* | **p-value** | **Beta**  **(95% CI)***^1^* | **p-value** | **Beta**  **(95% CI)***^1^* | **p-value** | **Beta**  **(95% CI)***^1^* | **p-value** | **Beta**  **(95% CI)***^1^* | **p-value** |
| DMLC Total | 0.30   (0.08 to 0.51) | **0.008** | 0.15   (-0.11 to 0.41) | 0.263 | 0.19   (-0.10 to 0.48) | 0.194 | 0.10   (-0.06 to 0.25) | 0.209 | 0.15   (-0.07 to 0.37) | 0.182 | -0.23   (-0.52 to 0.05) | 0.107 |
| DMLC Memory | 0.30   (0.09 to 0.51) | **0.007** | 0.16   (-0.10 to 0.42) | 0.212 | 0.12   (-0.17 to 0.41) | 0.417 | 0.08   (-0.08 to 0.23) | 0.32 | 0.10   (-0.12 to 0.32) | 0.377 | -0.20   (-0.49 to 0.08) | 0.152 |
| DMLC Not-Memory | 0.24   (0.02 to 0.46) | **0.035** | 0.10   (-0.17 to 0.36) | 0.466 | 0.28   (0.00 to 0.57) | 0.052 | 0.12   (-0.04 to 0.27) | 0.127 | 0.21   (-0.01 to 0.43) | 0.055 | -0.24   (-0.53 to 0.04) | 0.094 |
| CCI-20 Total | 0.17   (-0.06 to 0.40) | 0.148 | -0.07   (-0.34 to 0.20) | 0.597 | -0.18   (-0.47 to 0.12) | 0.235 | 0.03   (-0.13 to 0.19) | 0.749 | 0.19   (-0.03 to 0.41) | 0.095 | -0.35   (-0.63 to -0.07) | **0.015** |
| CCI-20 Memory | 0.15   (-0.08 to 0.37) | 0.197 | -0.09   (-0.35 to 0.18) | 0.521 | -0.18   (-0.47 to 0.11) | 0.226 | 0.01   (-0.15 to 0.16) | 0.936 | 0.20   (-0.01 to 0.42) | 0.067 | -0.32   (-0.60 to -0.04) | **0.024** |
| CCI-20 Not Memory | 0.17   (-0.07 to 0.40) | 0.162 | -0.03   (-0.30 to 0.25) | 0.854 | -0.13   (-0.43 to 0.17) | 0.389 | 0.06   (-0.10 to 0.22) | 0.477 | 0.11   (-0.12 to 0.34) | 0.337 | -0.32   (-0.60 to -0.03) | **0.032** |
| **Cognitively Unimpaired (N = 162)** | | | | | | | | | | | | |
| **SCC Metric** | Aβ40 | | Aβ42 | | GFAP | | NfL | | p-tau181 | | Aβ42 / Aβ40 | |
|  | **Beta**  **(95% CI)***^1^* | **p-value** | **Beta**  **(95% CI)***^1^* | **p-value** | **Beta**  **(95% CI)***^1^* | **p-value** | **Beta**  **(95% CI)***^1^* | **p-value** | **Beta**  **(95% CI)***^1^* | **p-value** | **Beta**  **(95% CI)***^1^* | **p-value** |
| DMLC Total | -0.01   (-0.15 to 0.13) | 0.866 | 0.02   (-0.13 to 0.16) | 0.828 | -0.08   (-0.24 to 0.09) | 0.352 | 0.00   (-0.14 to 0.14) | 0.982 | 0.24   (0.08 to 0.40) | **0.003** | 0.03   (-0.10 to 0.17) | 0.618 |
| DMLC Memory | 0.01   (-0.13 to 0.15) | 0.907 | 0.04   (-0.11 to 0.18) | 0.626 | -0.03   (-0.20 to 0.13) | 0.677 | 0.06   (-0.08 to 0.19) | 0.418 | 0.29   (0.14 to 0.45) | **3.32E-04** | 0.03   (-0.10 to 0.17) | 0.634 |
| DMLC Not-Memory | -0.03   (-0.17 to 0.11) | 0.63 | -0.01   (-0.15 to 0.13) | 0.868 | -0.11   (-0.27 to 0.05) | 0.177 | -0.07   (-0.20 to 0.07) | 0.346 | 0.12   (-0.04 to 0.28) | 0.143 | 0.03   (-0.11 to 0.17) | 0.686 |
| CCI-20 Total | -0.10   (-0.25 to 0.05) | 0.199 | -0.04   (-0.20 to 0.11) | 0.589 | 0.02   (-0.16 to 0.20) | 0.799 | 0.01   (-0.14 to 0.16) | 0.909 | 0.04   (-0.14 to 0.22) | 0.642 | 0.10   (-0.05 to 0.25) | 0.193 |
| CCI-20 Memory | -0.10   (-0.25 to 0.05) | 0.198 | -0.02   (-0.18 to 0.13) | 0.747 | 0.02   (-0.16 to 0.19) | 0.844 | 0.01   (-0.13 to 0.16) | 0.862 | 0.06   (-0.11 to 0.24) | 0.481 | 0.13   (-0.02 to 0.28) | 0.079 |
| CCI-20 Not Memory | -0.07   (-0.23 to 0.08) | 0.351 | -0.06   (-0.22 to 0.09) | 0.421 | 0.03   (-0.15 to 0.21) | 0.766 | 0.00   (-0.15 to 0.15) | 0.979 | -0.01   (-0.19 to 0.17) | 0.917 | 0.01   (-0.14 to 0.16) | 0.912 |
| *^1^* CI = Confidence Interval | | | | | | | | | | | | |

Supplemental Table 3.3: Associations between each SCC Metric and Biomarker stratified by Race / Ethnicity (NH-White & NH-Black) adjusted for the following covariates: age, gender, cognitive status (MCI / CU), years of education, and depression (as measured by the Geriatric Depression Scale). Bold indicates significant association at $\alpha=0.05$

| **Non-Hispanic Black (N = 88)** | | | | | | | | | | | | |
| --- | --- | --- | --- | --- | --- | --- | --- | --- | --- | --- | --- | --- |
| **SCC Metric** | Aβ40 | | Aβ42 | | GFAP | | NfL | | p-tau181 | | Aβ42 / Aβ40 | |
|  | **Beta**  **(95% CI)***^1^* | **p-value** | **Beta**  **(95% CI)***^1^* | **p-value** | **Beta**  **(95% CI)***^1^* | **p-value** | **Beta**  **(95% CI)***^1^* | **p-value** | **Beta**  **(95% CI)***^1^* | **p-value** | **Beta**  **(95% CI)***^1^* | **p-value** |
| DMLC Total | 0.05   (-0.12 to 0.22) | 0.588 | 0.01   (-0.17 to 0.20) | 0.88 | 0.10   (-0.12 to 0.32) | 0.362 | 0.00   (-0.16 to 0.15) | 0.955 | 0.16   (-0.04 to 0.36) | 0.111 | -0.06   (-0.25 to 0.13) | 0.556 |
| DMLC Memory | 0.05   (-0.12 to 0.22) | 0.551 | 0.03   (-0.16 to 0.21) | 0.76 | 0.11   (-0.11 to 0.33) | 0.333 | -0.03   (-0.19 to 0.13) | 0.707 | 0.16   (-0.03 to 0.36) | 0.099 | -0.04   (-0.23 to 0.15) | 0.672 |
| DMLC Not-Memory | 0.03   (-0.14 to 0.20) | 0.714 | -0.01   (-0.19 to 0.17) | 0.919 | 0.07   (-0.15 to 0.29) | 0.521 | 0.04   (-0.12 to 0.19) | 0.658 | 0.12   (-0.08 to 0.32) | 0.236 | -0.07   (-0.26 to 0.12) | 0.466 |
| CCI-20 Total | -0.02   (-0.21 to 0.16) | 0.794 | -0.10   (-0.30 to 0.09) | 0.306 | -0.07   (-0.30 to 0.17) | 0.577 | -0.04   (-0.21 to 0.13) | 0.647 | 0.17   (-0.04 to 0.38) | 0.106 | -0.07   (-0.27 to 0.14) | 0.518 |
| CCI-20 Memory | -0.06   (-0.24 to 0.12) | 0.498 | -0.13   (-0.32 to 0.06) | 0.171 | -0.05   (-0.28 to 0.18) | 0.665 | -0.06   (-0.22 to 0.10) | 0.467 | 0.20   (-0.01 to 0.40) | 0.057 | -0.04   (-0.24 to 0.16) | 0.684 |
| CCI-20 Not Memory | 0.06   (-0.13 to 0.24) | 0.549 | -0.01   (-0.21 to 0.19) | 0.913 | -0.08   (-0.32 to 0.16) | 0.514 | 0.01   (-0.16 to 0.19) | 0.865 | 0.07   (-0.15 to 0.29) | 0.509 | -0.10   (-0.31 to 0.11) | 0.348 |
| **Non-Hispanic White (N = 113)** | | | | | | | | | | | | |
| **SCC Metric** | Aβ40 | | Aβ42 | | GFAP | | NfL | | p-tau181 | | Aβ42 / Aβ40 | |
|  | **Beta**  **(95% CI)***^1^* | **p-value** | **Beta**  **(95% CI)***^1^* | **p-value** | **Beta**  **(95% CI)***^1^* | **p-value** | **Beta**  **(95% CI)***^1^* | **p-value** | **Beta**  **(95% CI)***^1^* | **p-value** | **Beta**  **(95% CI)***^1^* | **p-value** |
| DMLC Total | 0.11   (-0.08 to 0.29) | 0.25 | 0.07   (-0.11 to 0.26) | 0.438 | -0.06   (-0.28 to 0.15) | 0.56 | 0.04   (-0.13 to 0.21) | 0.614 | 0.28   (0.08 to 0.47) | **0.005** | -0.07   (-0.25 to 0.11) | 0.451 |
| DMLC Memory | 0.15   (-0.04 to 0.33) | 0.116 | 0.10   (-0.08 to 0.29) | 0.276 | -0.03   (-0.25 to 0.19) | 0.796 | 0.12   (-0.05 to 0.29) | 0.166 | 0.31   (0.12 to 0.50) | **0.002** | -0.09   (-0.27 to 0.10) | 0.353 |
| DMLC Not-Memory | 0.04   (-0.15 to 0.22) | 0.703 | 0.02   (-0.16 to 0.20) | 0.838 | -0.09   (-0.31 to 0.12) | 0.392 | -0.06   (-0.23 to 0.11) | 0.495 | 0.17   (-0.02 to 0.37) | 0.078 | -0.04   (-0.22 to 0.15) | 0.691 |
| CCI-20 Total | -0.07   (-0.26 to 0.12) | 0.459 | -0.04   (-0.23 to 0.15) | 0.694 | -0.07   (-0.29 to 0.15) | 0.54 | 0.03   (-0.14 to 0.21) | 0.703 | -0.05   (-0.26 to 0.15) | 0.623 | 0.09   (-0.10 to 0.28) | 0.35 |
| CCI-20 Memory | -0.07   (-0.25 to 0.12) | 0.492 | -0.03   (-0.22 to 0.15) | 0.717 | -0.09   (-0.31 to 0.13) | 0.406 | 0.04   (-0.13 to 0.22) | 0.623 | -0.05   (-0.25 to 0.15) | 0.622 | 0.08   (-0.10 to 0.27) | 0.369 |
| CCI-20 Not Memory | -0.06   (-0.25 to 0.13) | 0.547 | -0.03   (-0.22 to 0.16) | 0.743 | 0.00   (-0.22 to 0.23) | 0.967 | 0.00   (-0.17 to 0.18) | 0.979 | -0.03   (-0.24 to 0.17) | 0.747 | 0.07   (-0.12 to 0.25) | 0.489 |
| *^1^* CI = Confidence Interval | | | | | | | | | | | | |
